# Effects of combination therapy with vildagliptin and valsartan in a mouse model of type 2 diabetes

**DOI:** 10.1186/1475-2840-12-160

**Published:** 2013-11-04

**Authors:** Katsutoshi Miyagawa, Tatsuya Kondo, Rieko Goto, Rina Matsuyama, Kaoru Ono, Sayaka Kitano, Shuji Kawasaki, Motoyuki Igata, Junji Kawashima, Takeshi Matsumura, Hiroyuki Motoshima, Eiichi Araki

**Affiliations:** 1 Department of Metabolic Medicine, Faculty of Life Sciences, Kumamoto University, 1-1-1 Honjo, Chuo Ward, Kumamoto 860-8556, Japan

**Keywords:** DPP-4 inhibitor, ARB, Insulin resistance, Inflammation, Hepatic steatosis

## Abstract

**Background:**

Dipeptidyl peptidase-4 (DPP-4) inhibitors modulate incretin hormones and exert anti-diabetic effects in type 2 diabetes mellitus. Treatment with angiotensin II type 1 receptor blockers (ARB) is a proven successful intervention for hypertension with type 2 diabetes. The present study investigated the combined effects of the DPP-4 inhibitor vildagliptin and the ARB valsartan in a mouse model of type 2 diabetes.

**Methods:**

C57BL/6 J mice fed with high-fat diet (HFD) or db/db mice were treated with placebo, phloridzin (PHZ), vildagliptin alone (ViL), valsartan alone (VaL) or ViL with VaL (ViLVaL) for 8 weeks.

**Results:**

Glucose metabolism was improved in response to PHZ, ViL and ViLVaL in both HFD and db/db mice. Upon glucose challenge, ViLVaL showed the greatest suppression of blood glucose excursions, with increased insulin secretion, in db/db mice. ViLVaL treatment also showed an improvement of insulin sensitivity in db/db mice. Serum inflammatory cytokines were significantly decreased, and adiponectin was highest, in the ViLVaL group. ViLVaL improved insulin signaling and attenuated stress signaling in liver with amelioration of hepatic steatosis due to activated fatty acid oxidation in db/db mice. Furthermore, immunohistochemical analysis of the pancreas revealed that the combination treatment resulted in an increased expression of insulin and PDX-1, and increased insulin content.

**Conclusions:**

The combination therapy of ViL and VaL improves both pancreatic beta-cell function and insulin sensitivity, with a reduction of the inflammatory and cell stress milieu in mouse models of T2DM. Our results suggest that this combination therapy exerts additive or even synergistic benefits to treat T2DM.

## Background

Type 2 diabetes is a major global health problem associated with high morbidity and excessive mortality. Because the prevalence of type 2 diabetes is rapidly increasing and current treatments are not sufficient to satisfactorily control the disease in the majority of patients, better treatments are still required [1]. Pancreatic beta-cell dysfunction and insulin resistance are the two major pathophysiological features of type 2 diabetes [2]. Hence, to maintain normoglycemia, the most desirable treatment for type 2 diabetes should target improvement of beta-cell dysfunction and insulin resistance simultaneously.

Diabetic micro- and macrovascular complications are at least partly dependent on dysglycemia itself, which has two main components: chronic sustained hyperglycemia and post-prandial glycemic fluctuations. Both components lead to diabetic complications through several major mechanisms, including activation of oxidative stress and increased chronic inflammatory activity [3]. Oxidative stress has been highly and positively correlated with glycemic variability over a daily period as assessed from the mean amplitude of glycemic excursions (MAGE). In this context, tight glycemic control, as well as suppression of chronic inflammation and cellular stress, is required to achieve ideal treatment of type 2 diabetes.

Glucagon-like peptide 1 (GLP-1) is a gut-derived incretin hormone that stimulates insulin and suppresses glucagon, inhibits gastric emptying, and reduces appetite and food intake. Because the action of incretin is attenuated in type 2 diabetics [4], exogenous GLP-1 administration improves glycemic control [5]. Therapeutic approaches for enhancing incretin action include degradation-resistant GLP-1 receptor agonists and inhibitors of dipeptidyl peptidase-4 activity [6]. DPP-4 inhibitors including vildagliptin have been reported to amplify beta-cell mass and improve its function in a number of *in vitro* and i*n vivo* studies [7-10]. A significant and sustained increase in active GLP-1 with suppression of glucagon in response to vildagliptin has been reported in type 2 diabetes [11]. Vildagliptin improves blood glucose stability in a dose-independent manner [12]. Furthermore, vildagliptin may be superior as a means to control MAGE and mean 24 h blood glucose compared with other DPP-4 inhibitors [13]. Blunting glucose fluctuations contributes to reducing oxidative stress and inflammation in diabetics [14]. These results together suggest that, among DPP-4 inhibitors, vildagliptin may have the best potential to achieve total glycemic control and minimize glucose fluctuations.

Patients with type 2 diabetes frequently experience complications such as hypertension. Inhibition of the renin-angiotensin system (RAS), especially through the use of angiotensin II type 1 receptor blockers (ARB) such as valsartan, was reported to have a preventive effect on type 2 diabetes [15,16]. In particular, a lower incidence of newly diagnosed diabetes was confirmed in response to valsartan treatment [17]. Recent studies have suggested that valsartan increases glucose-stimulated insulin secretion (GSIS) and insulin sensitivity in normotensive subjects with impaired glucose tolerance (IGT) [18]. The prospective NAVIGATOR trial showed that treatment with valsartan reduced type 2 diabetes incidence by 14% in subjects with IGT [19]. ARB treatment was shown to improve GSIS and insulin biosynthesis, and delay the onset of diabetes, in db/db mice [20]. These observations suggest that a combination of the DPP-4 inhibitor vildagliptin with the ARB valsartan may exert beneficial effects in the treatment of type 2 diabetes through complementary actions.

## Materials and methods

### Animals

All animals, obtained from CLEA (CLEA Japan Inc., Tokyo, Japan), were housed in the Center for Animal Resources and Development of Kumamoto University. The experimental procedures were approved by the Animal Experimentation Ethics Committee of Kumamoto University (B23-178, B24-128).

### Diet and treatment protocol

C57BL/6 mice were fed High Fat Diet 32 (HFD group) (CLEA), and db/db mice were fed Rodent Diet CE-2 (db/db group) (CLEA). We had a total of 66 4-week-old db/db mice and 66 6-week-old C57BL/6 J mice (2 weeks of HFD in advance). The first 60 mice of each strain were randomly divided into the following five groups, and the remaining 6 mice of each strain were then randomly assigned to the final 3 groups. All mice received their respective treatments for 8 weeks:

(1) CT: placebo treatment (n = 12), (2) PHZ: Phloridzin treatment (0.5% in diet) (n = 12), (3) ViL: Vildagliptin treatment (1 mg/kg/day in drinking water) (n = 14), (4) VaL: Valsartan treatment (10 mg/kg/day in drinking water) (n = 14), (5) ViLVaL: Vildagliptin (1 mg/kg/day) and Valsartan treatment (10 mg/kg/day) (n = 14). The levels of ViL and VaL used in this study were chosen based on previous studies [21,22] and represent relevant physiological doses for a human clinical situation.

### Measurements of biochemical markers

Blood glucose was measured using a Glutest Neo (Sanwa Chemical Co., Nagoya, Japan). The concentration of insulin was measured using an insulin ELISA kit (Shibayagi, Gunma, Japan). Serum C-reactive protein (CRP; Life Diagnostics Inc., West Chester, PA), tumor necrosis factor (TNF)-α (Invitrogen, Carlsbad, CA), monocyte chemoattractant protein (MCP)-1 (Invitrogen), interleukin (IL)-6 (R&D Systems Inc., Minneapolis, MN), leptin (Crystal Chem Inc., Chicago, IL) and adiponectin (Otsuka Pharmaceutical, Tokyo, Japan) levels were measured using the appropriate ELISA kits.

### Intra peritoneal glucose tolerance test (GTT) and intraperitoneal insulin tolerance test (ITT)

After 8 weeks of treatment, GTT (2.0 g/kg) and ITT (1 U/kg for HFD or 2 U/kg for db/db) were performed following an intra-peritoneal injection of glucose or insulin, as previously described [23].

### Pancreatic insulin content

The pancreas was rapidly removed, homogenized, and extracted in acid ethanol overnight at 4°C. The insulin content was measured using a Mouse Insulin ELISA Kit (Shibayagi) [24].

### In situ measurement of nitrotyrosine

Liver tissue was dissected and immediately frozen in liquid nitrogen. After homogenization, the content of nitrotyrosine was measured using NWLSS™ Nitrotyrosine ELISA test kits (Northwest Life Science Specialties, LLC).

### Western blot analysis

The liver and muscle tissues were dissected 5 min after injection with or without insulin (5 U/mice) via the inferior vena cava and immediately frozen in liquid nitrogen. Western blotting was performed as described previously [24] using antibodies purchased from Cell Signaling Technology (CST; Beverly, MA, USA), except for anti-α-tubulin clone DM1A, which was purchased from Millipore (Billerica, MA, USA).

### Immunohistochemistry

Immunohistochemical analysis was performed as described previously [24]. Anti-insulin antibody (diluted 1:200, Santa Cruz (SC) Biotechnology Inc., Santa Cruz, CA), anti-glucagon antibody (diluted 1:100, SIGMA-ALDRICH), anti-PDX-1 antibody (diluted 1:100, CHEMICON International Inc., Temecula, CA USA), anti-IRS2 antibody (diluted 1:100, SC), anti-NF-κB p65 antibody (diluted 1:100, CST) and anti-HSP72 antibody (diluted 1:100, Stressgen Biotechnologies, Victoria, BC, Canada) were used.

### Morphometric analysis

The area of insulin positive beta-cells relative to the total area of pancreatic tissue was calculated using Image J image analysis software (Version 1.61; http://rsbweb.nih.gov/ij/), using 18 sections (i.e., six sections from three different areas of the pancreas) for each group of mice.

### Statistical analysis

All data are shown as the mean ± S.E.M. Comparisons of multiple means were performed using two-way repeated measures analysis of variance (for homogeneous variance) as indicated. Multiple comparisons between groups were performed using an analysis of variance followed by Tukey’s post-hoc test. A value of p < 0.05 was considered statistically significant.

## Results

### Combination treatment with vildagliptin and valsartan decreases blood glucose

**Figure 1:**
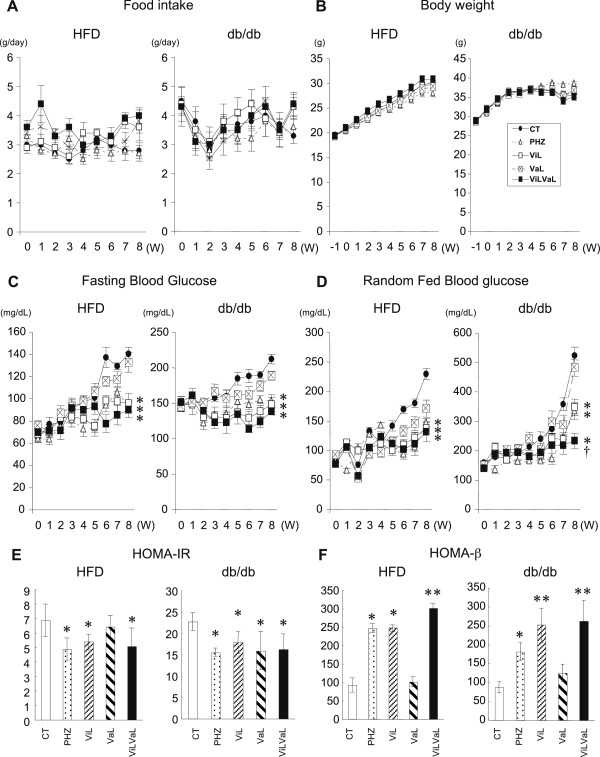
**Combination treatment with vildagliptin and valsartan decreases blood glucose in HFD and db/db mice.** Weekly food intake **(A)**, body weight increments **(B)**, fasting **(C)** and random-fed blood glucose **(D)** were measured during each treatment in HFD or db/db mice. HOMA-IR and HOMA-beta were calculated using fasting blood glucose and insulin levels after 8 weeks of treatment **(E and F)**. Values represent mean ± SEM, n = 12 – 14. *p < 0.05, **p < 0.01 compared with the CT group. †p < 0.05 compared with the ViL group.

After 8 weeks of treatment, food intake (Figure 1A) and body weight (Figure 1B) were unaltered in all groups. Fasting blood glucose levels were significantly decreased in PHZ (HFD: -33.3%, p = 0.027; db/db: -35.7%, p = 0.019), ViL (HFD: -31.6%, p = 0.041; db/db: -39.8%, p = 0.032) and ViLVaL (HFD: -35.8%, p = 0.012; db/db: -42.6%, p = 0.028) interventions compared with the corresponding CT groups (HFD: CT: 140.3 ± 6.3 mg/dL, db/db: CT: 229.5 ± 9.6 mg/dL) after 8 weeks of treatment (Figure 1C). There were similar changes in random-fed glucose levels in the PHZ (HFD: -27.8%, p = 0.044; db/db: -36.0%, p = 0.021), ViL (HFD: -30.1%, p = 0.040; db/db: -33.6%, p = 0.018) and ViLVaL (HFD: -34.9%, p = 0.029; db/db: -55.5%, p = 0.005, Figure 1D) groups compared with CT (HFD: CT: 212.5 ± 6.8 mg/dL, db/db: CT: 523.2 ± 29.8 mg/dL).

The VaL group did not show a significant decrease in either fasting or random-fed glucose levels (Figure 1C and D). The most profound suppression of random glucose levels was observed in ViLVaL-treated db/db animals compared with ViL-treated db/db animals (-33.0%, p = 0.036), suggesting that the combination therapy may have additional effects on glucose lowering.

After 8 weeks of treatment, homeostatic model assessment insulin resistance (HOMA-IR) of HFD mice was significantly decreased in PHZ (CT: 6.8 ± 1.1 PHZ: -29.0%, p = 0.033), ViL (-21.2%, p = 0.041) and ViLVaL (-26.0%, p = 0.015) compared with CT mice (Figure 1E). HOMA-IR of db/db mice was significantly reduced in all treatment groups compared with the control group (CT: 22.7 ± 2.1. PHZ: -31.7%, p = 0.046; ViL: -21.0%, p = 0.032; VaL: -30.0%, p = 0.028; ViLVaL: -28.2%, p = 0.024) (Figure 1E).

HOMA-beta in HFD showed a significant increase in the PHZ (CT: 91.9 ± 19.2 PHZ: + 168.1%, p = 0.02), ViL (+170.0%, p = 0.031) and ViLVaL (+228.3%, p = 0.018) groups compared with CT animals (Figure 1F). HOMA-beta in db/db was also upregulated in PHZ (CT: 86.5 ± 14.2. PHZ: +109.1%, p = 0.016), ViL (+190.9%, p = 0.003) and ViLVaL (+202.7%, p = 0.007) groups compared with the CT group (Figure 1F). These data indicate that PHZ, ViL and ViLVaL treatment improved blood glucose, insulin resistance and basal insulin secretion in both groups of mice.

### Combination treatment with vildagliptin and valsartan improves both insulin resistance and insulin secretion

**Figure 2:**
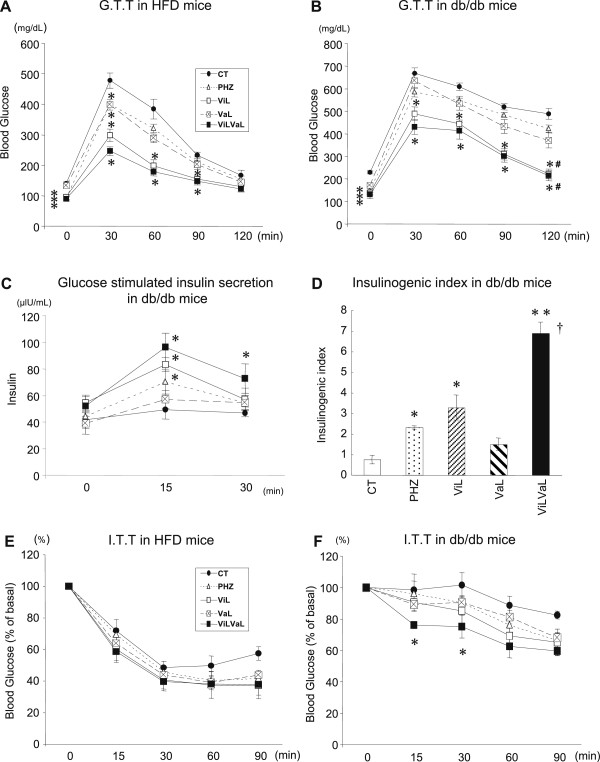
**Combination treatment with vildagliptin and valsartan improves both insulin resistance and insulin secretion in HFD and db/db mice.** Glucose tolerance was evaluated by GTT in HFD **(A)** or db/db **(B)** mice. Glucose-stimulated insulin secretion **(C)** and the insulinogenic index **(D)** in response to the GTT are shown. Insulin sensitivity was evaluated by ITT in HFD **(E)** or db/db **(F)** mice. Values represent mean ± SEM, n = 12 – 14. *p < 0.05, **p < 0.01 compared with the CT group. #p < 0.05 compared with the VaL group. †p < 0.05 compared with the ViL group.

To further examine glucose homeostasis in these animals, we performed GTT with measurements of insulin secretion, and ITT. After 8 weeks of treatments, GTT in HFD mice showed that the blood glucose levels in all treatment groups 30 min after injection were significantly lower than those in the CT group (Figure 2A). Glucose levels in the ViL and ViLVaL-treated groups at 60 and 90 min were also significantly lower than those in the CT group (Figure 2A).

GTT in db/db mice showed that the blood glucose levels in ViL and ViLVaL-treated groups 30, 60, 90 and 120 min after injection were significantly lower than those in the CT group (Figure 2B). At 120 min after injection, ViL and ViLVaL treatment groups displayed a significant decrease in blood glucose levels compared with the VaL group (Figure 2B).

Serum insulin responses during GTT in db/db mice were improved in PHZ, ViL and ViLVaL mice at 15 min and in ViLVaL mice at 30 min (Figure 2C). Insulinogenic index (I.I.) during GTT in db/db was also improved in the PHZ, ViL and ViLVaL groups (Figure 2D). I.I. in the ViLVaL-treated group was significantly increased even compared with ViL monotherapy (Figure 2D).

The insulin sensitivity estimated by ITT in HFD mice was unchanged in all treated groups (Figure 2E). In db/db mice, only ViLVaL-treated mice showed significantly lower glucose levels at 15 and 30 min after insulin injection (Figure 2F).

### Combination ViLVaL treatment decreases CRP, TNF-α, IL-6 and MCP-1, and increases adiponectin levels

Dysregulation of inflammatory cytokines and adipokines is an important pathophysiological feature of type 2 diabetes, and this was investigated 8 weeks after treatment in HFD and db/db mice (Table 1).

**Table 1:** Plasma inflammatory cytokines and adipokines in HFD and db/db mice treated with corresponding interventions

**HFD mice**					
	**CT**	**PHZ**	**ViL**	**VaL**	**ViLVaL**
CRP (ng/mL)	51.6 ± 6.1	46.1 ± 4.0 (0.18)	39.7 ± 3.4* (0.027)	44.0 ± 4.9* (0.043)	35.9 ± 2.2** (0.0042)
TNF-α (pg/mL)	12.9 ± 1.6	12.7 ± 1.5 (0.53)	10.9 ± 0.7* (0.023)	10.0 ± 0.6* (0.029)	9.0 ± 0.6* (0.011)
IL-6 (pg/mL)	11.1 ± 1.1	10.8 ± 1.0 (0.13)	9.8 ± 0.6 (0.09)	10.8 ± 0.5 (0.07)	8.7 ± 0.5* (0.032)
MCP-1 (pg/mL)	76.4 ± 7.3	73.5 ± 5.6 (0.21)	67.8 ± 4.5 (0.64)	59.4 ± 5.0* (0.033)	45.7 ± 3.6** ^†^ (0.003)
Leptin (ng/mL)	15.6 ± 1.1	15.3 ± 1.0 (0.32)	15.0 ± 0.9 (0.53)	15.1 ± 0.5 (0.074)	15.0 ± 0.09 (0.09)
Adiponectin (μg/dL)	8.7 ± 0.9	8.4 ± 0.7 (0.28)	9.8 ± 0.5* (0.031)	8.8 ± 0.6 (0.09)	10.8 ± 0.5** (0.004)
**db/db mice**					
	**CT**	**PHZ**	**ViL**	**VaL**	**ViLVaL**
CRP (ng/mL)	70.1 ± 4.1	55.1 ± 2.3* (0.039)	46.4 ± 3.9* (0.012)	53.3 ± 4.9* (0.024)	38.5 ± 3.6** (0.007)
TNF-α (pg/mL)	18.6 ± 1.0	15.6 ± 1.6 (0.24)	11.8 ± 0.7** (0.002)	10.1 ± 0.5** (0.0042)	10.2 ± 0.5** (0.0002)
IL-6 (pg/mL)	17.1 ± 0.9	14.3 ± 0.8 (0.11)	13.2 ± 0.8* (0.045)	12.1 ± 0.8* (0.043)	11.9 ± 0.8* (0.039)
MCP-1 (pg/mL)	118.7 ± 8.6	105.6 ± 7.3 (0.43)	73.5 ± 3.9** (0.0002)	68.2 ± 5.6** (0.003)	56.4 ± 3.1** (0.0005)
Leptin (ng/mL)	23.2 ± 1.4	22.6 ± 1.1 (0.18)	22.1 ± 1.1 (0.26)	23.5 ± 0.7 (0.31)	23.1 ± 0.7 (0.85)
Adiponectin (μg/dL)	6.1 ± 0.6	7.2 ± 0.5 (0.07)	7.7 ± 0.5* (0.0015)	6.9 ± 0.4 (0.078)	7.15 ± 0.5* (0.0043)

Values represent mean ± SEM, n = 12 – 14.

*p < 0.05, **p < 0.01 compared with the CT group. ^†^p < 0.05 compared with the ViL group.

The numbers in parenthesis indicate p values.

In HFD mice, plasma CRP levels were significantly decreased in ViL, VaL and ViLVaL-treated mice compared with CT mice. TNF-α levels were also significantly reduced in ViL, VaL and ViLVaL-treated mice compared with CT mice. IL-6 was significantly decreased only in ViLVaL compared with CT mice. Plasma MCP-1 levels were significantly lower in the VaL and ViLVaL groups compared with the CT group. Interestingly, ViLVaL showed additional suppression of MCP-1 levels compared with ViL monotherapy. Plasma leptin levels were unaffected by any treatment. Plasma adiponectin levels were significantly increased in ViL and ViLVaL mice compared with CT mice, being highest in the ViLVaL group.

In db/db mice, similar trends were observed in inflammatory cytokines and adipokines. As a result, ViLVaL treatment group showed a strong improvement in inflammatory markers both in HFD and db/db mice.

### Combination therapy attenuates insulin resistance with alleviation of ER stress and oxidative stress in liver

**Figure 3:**
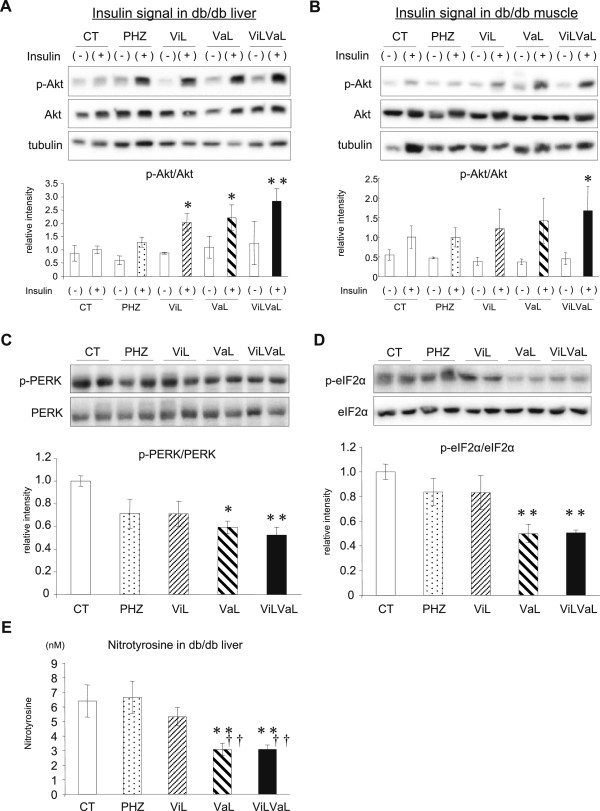
**Combination therapy attenuates insulin resistance with alleviation of ER stress and oxidative stress in liver.** Liver **(A)** or muscle **(B)** lysates were extracted 8 weeks after initiation of each treatment in db/db mice with or without 5 units of insulin stimulation through the inferior vena cava, and were analyzed by western blotting. PERK **(C)** and eIF2α phosphorylation **(D)** were also analyzed in liver samples without insulin stimulation. The amount of nitrotyrosine **(E)** was measured in liver of db/db mice. Values represent mean ± SEM, n = 6 – 8. *p < 0.05, **p < 0.01 significantly different from the CT group. ††p < 0.05 compared with the PHZ group.

To elucidate the molecular mechanism of whole-body insulin sensitization in mice treated with ViL and/or VaL, insulin signaling in db/db mouse liver and muscle was examined. Upon insulin stimulation *in vivo*, phosphorylation of Akt in the liver was increased in response to ViL (2.03 fold), VaL (2.21 fold) and ViLVaL (2.83 fold) treatment (Figure 3A), and was also increased in response to ViLVaL in muscle (1.68 fold, Figure 3B).

In order to clarify the improved insulin signaling in liver, endoplasmic reticulum (ER) stress signaling and oxidative stress markers were assessed. Phosphorylation of PKR-like ER kinase (PERK) was significantly decreased in the VaL (-40.7 ± 5.6%, p = 0.019) and ViLVaL (-47.6 ± 6.8%, p = 0.006) treatment groups compared with CT mice (Figure 3C). Phosphorylation of eukaryotic translation initiation factor 2A (eIF2α) was also decreased in the VaL (-50.0 ± 7.6%, p = 0.005) and ViLVaL (-49.4 ± 2.3%, p = 0.006) treatment groups (Figure 3D).

The oxidative stress marker nitrotyrosine was significantly decreased in VaL and ViLVaL treatment groups compared with the CT and PHZ groups (CT: 6.40 ± 1.10 nM; PHZ: +3.9%, p = 0.999; ViL: -16.7%, p = 0.818; VaL: -52.0%, p = 0.025 (p = 0.014 vs. PHZ); ViLVaL: -54.0%, p = 0.024 (p = 0.014, vs. PHZ: Figure 3E)). VaL and ViLVaL treatment groups showed improved insulin signaling accompanied by alleviation of ER stress and oxidative stress.

### Combination therapy improves hepatic lipid accumulation

**Figure 4:**
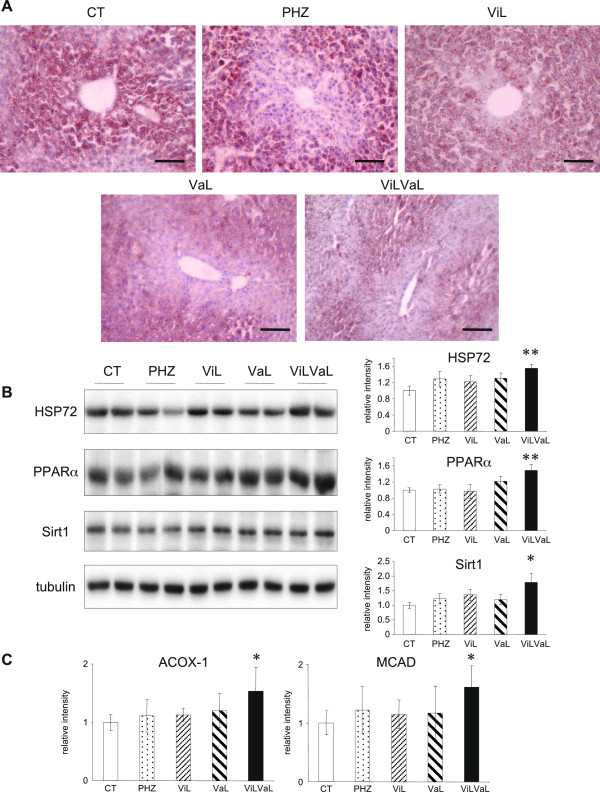
**ViLVaL combination therapy improves hepatic lipid accumulation.** Hepatic steatosis was evaluated by Oil Red O staining of liver tissues of db/db mice. Scale bars indicate 100 μm at × 200 magnification **(A)**. Liver extracts were subjected to western blotting for HSP72, PPARα and Sirt1 **(B)**. Hepatic lipid oxidation was evaluated by measuring *Acox1* and *Mcad* mRNA expression. Values represent mean ± SEM, n = 6 – 8. *p < 0.05, **p < 0.01 compared with the CT group **(C)**.

Hepatic steatosis was examined by oil red O staining in liver of db/db mice. ViL or VaL alone showed a slight improvement of fatty liver, and there was a marked reduction of lipid accumulation in response to ViLVaL compared with the CT or PHZ groups (Figure 4A).

To further evaluate the molecular mechanisms of hepatic lipid reduction, the expression of heat shock protein (HSP)72, peroxisome proliferator-activated receptor (PPAR)α and sirtuin1 (Sirt1) were evaluated by western blotting, because these molecules are involved in insulin sensitivity and lipid metabolism in liver [23,25-27]. HSP72, PPARα and Sirt1 were significantly increased in the ViLVaL group compared with all other groups (Figure 4B), which was consistent with the decrease in lipid accumulation in liver.

In order to evaluate fatty acid beta-oxidation in liver, mRNA levels of peroxisomal acyl-CoA oxidase 1 (*Acox1*) and medium chain acyl-CoA dehydrogenase (*Mcad*) were measured. *Acox1*, the first enzyme of the beta-oxidation pathway, was 1.54 times higher compared with control (p = 0.041, Figure 4C). *Mcad*, which is responsible for initial dehydrogenation step in the beta-oxidation cycle, was also increased 1.61 times compared with control (p = 0.03, Figure 4C).

### Combination therapy contributes to maintenance of islet morphology and increases the pancreatic insulin content

**Figure 5:**
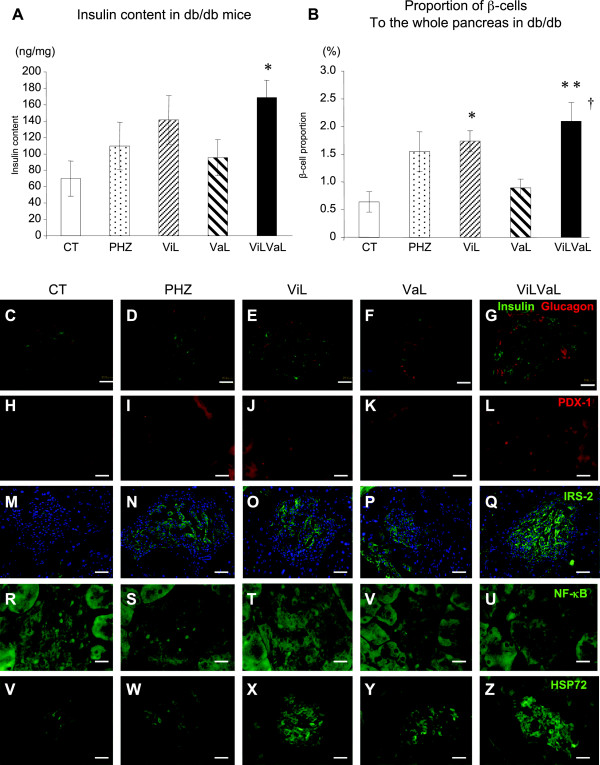
**ViLVaL combination therapy contributes to maintenance of islet morphology and increases the insulin content of the pancreas.** Insulin content was measured in pancreatic extracts **(A)**. The proportion of beta-cells to the whole pancreas was calculated using insulin-stained pancreatic sections **(B)**. Sections of pancreas were immunostained for insulin with glucagon **(C to G)**, PDX-1 **(H to L)**, IRS-2 (with DAPI; **M** to **Q**), NF-κB p65 subunits **(R to U)** or HSP72 **(V to Z)**. Scale bars indicate 20 μm at × 400 magnification. Values represent mean ± SEM, n = 6 – 8. *p < 0.05, **p < 0.01 compared with the CT group. †p < 0.05 compared with the ViL group.

To identify the molecular mechanism of improved insulin secretion in db/db mice treated with ViLVaL, we analyzed the morphology of pancreatic islets using immunohistochemistry. After 8 weeks of treatments, pancreatic insulin content was significantly increased in the ViLVaL treatment group compared with the CT group (CT: 82.47 ± 20.76 ng/mg; PHZ: +56.4%, p = 0.667; ViL: +77.6%, p = 0.366; VaL: +30.9%, p = 0.946; ViLVaL: +118.8%, p = 0.032: Figure 5A).

The beta-cell area relative to whole pancreas was also significantly increased in the ViL and ViLVaL groups compared with the CT group (CT: 0.64 ± 0.18% vs. PHZ: 1.55 ± 0.36%, p = 0.081; ViL: 1.73 ± 0.19%, p = 0.025; VaL: 0.89 ± 0.16, p = 0.944; ViLVaL: 2.10 ± 0.34, p = 0.0018: Figure 5B). The combination therapy group also showed a significant increase in beta-cell area compared with ViL monotherapy (p = 0.011, Figure 5B).

Morphological analysis showed that immuno-stained insulin was low in CT islets, and this was increased in the ViL and ViLVaL groups (Figure 5C–G). Nuclear localization of pancreatic and duodenal homeobox (PDX)-1 was only sparsely detectable in CT islets, but was increased in the ViL and ViLVaL groups (CT: 1.93 ± 0.93/islet vs. PHZ: 3.73 ± 1.34/islet, p = 0.01; ViL: 10.07 ± 2.67/islet, p = 0.005; VaL: 4.53 ± 1.54/islet, p = 0.02; ViLVaL: 19.93 ± 4.17/islet, p = 0.0001: Figure 5H-L). Anti-apoptotic insulin receptor substrate (IRS)-2 positive area in islets was relatively small in CT mice and was increased in each of the treatment groups (Figure 5M–Q). Inflammatory signal detected by nuclear factor (NF)-κB p65 subunit nuclear accumulation was activated in CT islets, and was clearly lower in ViLVaL islets (CT: 9.53 ± 2.25/islet vs. PHZ: 7.33 ± 2.02/islet, p = 0.04; ViL: 2.60 ± 0.95/islet, p = 0.002; VaL: 2.93 ± 1.06/islet, p = 0.003; ViLVaL: 2.20 ± 0.98/islet, p = 0.0001: Figure 5R-U). The inducible HSP72 is also associated with cell protection in islets [24]. Immuno-positive HSP72 was low in CT islets and was increased in ViL, VaL and ViLVaL islets (Figure 5V–Z). These results indicate that the combination therapy protects beta-cells from inflammation and degradation in db/db mice.

## Discussion

The main finding of this study is that a combination therapy of vildagliptin and valsartan significantly magnified the beneficial effect of either monotherapy in diabetic mice.

### Insulin sensitizing effects

The ViLVaL combination improved insulin signaling mainly in the liver, with attenuation of ER stress and oxidative stress. As ViL alone shows only slight insulin sensitization, the additional effect of VaL in ameliorating stress signals in liver could explain this additional benefit. The RAS cascade is activated in obesity, metabolic syndrome and type 2 diabetes [28]. RAS activation inhibits insulin signaling through multiple mechanisms, including IRS-1 serine phosphorylation, an increase of reactive oxygen species (ROS) and activation of c-jun N-terminal kinase (JNK), or TNF-α activation [28,29]. Indeed, AT1R blockade with valsartan has been shown to suppress nicotinamide adenine dinucleotide phosphate (NA(D)PH) oxidase p22^phox^ and gp91^phox^[30], ER stress [31] and NF-κB activation [32], resulting in reduced oxidative stress and inflammation. In this study, valsartan ameliorated the inflammatory milieu in liver, resulting in enhancement of insulin action.

HSP72 is a major inducible HSP against several stresses such as heat, ischemia or hypoxia, which protects cells through JNK inhibition [25]. The HSP72 level is decreased in type 2 diabetes and restoration of HSP72 improves insulin resistance and glucose homeostasis in mice [23-26]. We observed significant HSP72 up-regulation in liver in response to the ViLVaL combination, suggesting this also contributes to insulin sensitization.

Vildagliptin alone modulates the expression of genes important for lipid metabolism [7]. GLP-1 signaling stimulates PPARα and Sirt1 in liver, which in turn enhances fatty acid oxidation and attenuates inflammatory signals. The improvement of hepatic steatosis and presumably inflammation is likely due to activation of GLP-1R on hepatocytes, because others have shown that exenatide stimulates hepatocyte expression of PPARα and PPARγ that improve hepatic fatty acid oxidation, lipid export, and insulin sensitivity [33]. We observed a significant increase of HSP72, PPARα and Sirt1 with amelioration of hepatic steatosis, particularly in the combined treatment group, suggesting that not only vildagliptin, but also valsartan may participate in activating this pathway. Moreover, hepatic fatty acid beta-oxidation, evaluated by assessment of *Acox1* and *Mcad* RNA levels, was enhanced by the combination treatment, although the lipogenic pathway was not altered (data not shown).

### Beta-cell protecting effects

Cellular stresses such as ER stress and/or oxidative stress are key components in the pathophysiology of type 2 diabetes. In fact, pancreatic beta-cell specific C/EBPbeta transgenic mice exhibit diabetes and decreased beta-cell mass associated with increased apoptosis, decreased proliferation, and aggravated ER stress, and these can be restored by oral administration of vildagliptin [34]. Treatment of rats with vildagliptin significantly increased insulin content and decreased inflammatory markers such as nitric oxide and TNF-α [35,36]. Chronic administration of vildagliptin in IRS-2 KO mice improved glucose metabolism and suppressed beta-cell apoptosis, suggesting that vildagliptin may also activate beta-cell proliferation independent of IRS-2 axis [37]. HFD-induced glucose intolerance and beta cell inflammation were both prevented by chronic treatment with vildagliptin [10]. We observed a beta-cell protective effect of ViLVaL in the islets of db/db mice. ViL alone increased the proportion of beta-cells but there was not a significant increase in insulin content. The ViLVaL combination significantly increased both insulin content and the proportion of beta-cells, as well as up-regulating PDX-1 and HSP72, and attenuating NF-κB. We also found that IRS-2 was up-regulated in the treated islets. This effect may also contribute to the protection of beta-cells from apoptosis. HSP72 can attenuate NF-κB nuclear translocation by inhibiting the inhibitor of κB (IκB)αphosphorylation [25]. This indicates that the combination therapy attenuates pancreatic local inflammation, thus leading to beta-cell protection.

### Attenuation of chronic inflammation

We observed a significant reduction of circulating inflammatory markers such as CRP, TNF-α, IL-6 and MCP-1, and an increase of adiponectin, in both HFD and db/db mice. The ViLVaL combination significantly and profoundly improved these markers, indicating that the systemic chronic inflammatory milieu is alleviated. These cytokines are mainly produced by adipose tissues and macrophages. The RAS blockade decreases adipocyte size and adipose tissue inflammatory markers, and improves glucose homeostasis in rodents [20,38,39]. Valsartan treatment also reduces abdominal adipocyte size and macrophage infiltration in IGT subjects. These findings suggest that interventions with ViLVaL may attenuate adipose tissue inflammation, thereby contributing to the improvement of whole body glucose homeostasis [40].

Serum DPP-4 levels correlate with adipocyte size and adipocytes potentially represent an important source of DPP-4 in obesity, which impairs insulin signaling [41]. Inhibiting DPP-4 by vildagliptin may directly influence the insulin resistant effect of circulating DPP-4 itself.

We have demonstrated anti-inflammatory benefits of ViL in diabetic mice, however some researchers have reported that ViL may not have cardio-protective effects in a rat model of myocardial infarction [42]. An underlying diabetic phenotype might be necessary to observe any cardio-protective effects of ViL through its anti-inflammatory potency. This possibility needs to be elucidated in further studies.

### The benefits of valsartan in combination with vildagliptin

The combination therapy of vildagliptin and valsartan in db/db mice leads to improvement of islet ROS production, apoptotic events, beta-cell mass, and whole body glucose homeostasis [22]. The expression of NAD(P)H oxidase subunits is also significantly decreased by a similar combination resulting in decreased ROS production with GLP-1 up-regulation [30]. The decrease of vascular oxidative stress and inflammatory reactions in response to the combination therapy is also more prominent than monotherapy with valsartan or vildagliptin analogues [30]. These data support our findings, and we have further described the attenuation of systemic and local inflammation, hepatic steatosis, ER stress and HSP72 induction. We also found beneficial effects on glucose metabolism in the PHZ group, but the beneftts on insulin sensitization, cell stress attenuation and inflammation were relatively small, suggesting that the combination of a DPP-4 inhibitor with an ARB may have advantageous effects in addition to just lowering glucose, which is the case with PHZ.

Although this combined therapy reduced inflammatory cytokines and increased adiponectin, attenuated ER stress, and increased insulin content and proportion of beta-cells, the effect of this combination on glucose metabolism was relatively modest in GTT. Because a DPP-4 inhibitor works in the food-ingested state, an intraperitoneal glucose challenge may have represented a lesser stimulation than food ingestion, and thus led to relatively smaller effects. It is also possible that a longer treatment period with ViLVaL may have led to more pronounced glucose improvement effects, because the differences in glucose levels between treatment groups appeared to increase with time.

In conclusion, the combination of vildagliptin and valsartan shows additive or even synergistic effects in insulin action, hepatic lipid oxidation and beta-cell protection mediated by attenuation of cellular stresses and systemic/local inflammation. This combination may be useful to ameliorate insulin resistance and beta-cell failure simultaneously, and could further delay vascular complications in type 2 diabetes patients.

## Abbreviations

DPP-4: Dipeptidyl peptidase-4; ARB: Angiotensin II type 1 receptor blockers; HFD: High-fat diet; MAGE: Mean amplitude of glycemic excursions; GLP-1: Glucagon-like peptide 1; RAS: Renin-angiotensin system; GSIS: Glucose-stimulated insulin secretion; IGT: Impaired glucose tolerance; CRP: C-reactive protein; TNF-α: Tumor necrosis factor-α; MCP-1: Monocyte chemoattractant protein-1.

## Competing interests

The authors declare that they have no competing interests.

## Authors’ contributions

KM generated data, analyzed the data and wrote the manuscript. TK designed the research, generated data, wrote the manuscript*,* and is the guarantor of this work and, as such, had full access to all the data in the study and takes responsibility for the integrity of the data and the accuracy of the data analysis. RG, RM, KO, SK, and SK generated data. MI, JK, TM, and HM contributed to discussions and reviewed the manuscript. EA is the guarantor of this work and, as such, had full access to all the data in the study and takes responsibility for the integrity of the data and the accuracy of the data analysis. All authors read and approved the final manuscript.
